# Longing for a sense of belonging—Somali immigrant adolescents’ experiences of their acculturation efforts in Sweden

**DOI:** 10.1080/17482631.2020.1784532

**Published:** 2020-12-09

**Authors:** Fatumo Osman, Abdikerim Mohamed, Georgina Warner, Anna Sarkadi

**Affiliations:** a School of Education, Health and Social Studies, Dalarna University, Falun, Sweden; b Child Health and Parenting (CHAP), Department of Public Health and Caring Sciences, Uppsala University, Uppsala, Sweden

**Keywords:** Acculturation, adolescents, exclusion, inclusion, migration, sense of belonging

## Abstract

**Purpose:** Research on Somali adolescent immigrants’ interactions with their new society and school context is pertinent to more deeply understand changes in their practices, values, and identity resulting from continued first-hand contact with the Swedish culture, known as acculturation, as this process has been shown to affect mental health. The aim of this study was to investigate Somali immigrant adolescents’ experiences of social inclusion and exclusion as well as their need for support in their efforts to acculturate into society and the school environment.**Method:** A qualitative explorative study was performed. Data were collected from six focus group discussions with 47 Somali immigrant adolescents living in Sweden. The data were analysed using thematic network analysis.**Results:** A global theme emerged from the analysis: longing for a sense of belonging. Two underpinning organising themes described the participants’ longing for a sense of belonging to society and school: experience of social exclusion andpathways of inclusion and acculturation. Each organising theme consisted of three or four basic themes.**Conclusion:** This study highlights several key considerations on how schools can help adolescents who have recently immigrated to Sweden achieve a sense of belonging.

## Introduction

Acculturation refers to changes in individual’s practices, values, and identity resulting from continued first-hand contact with another culture (Ward, 2001). It is not as simple as detaching from the home culture and adopting the cultural patterns of the new country, but is a complex multidimensional process that can affect the social functioning and psychological well-being of the individual (Gupta et al., 2013; Nguyen & Benet-Martinez, 2013; Yoon et al., 2013). Traditional acculturation theory adopted categorizing systems that place individuals into groups according to the strength of their orientation towards their home or settlement culture (Berry, 1980, 1997); however, this is problematic in that it oversimplifies the process by dichotomizing between cultures. More recent evaluation of acculturation allows acculturating individuals to express themselves in their own words (Ward, 2013). This heuristic approach can elucidate a richer understanding of the acculturation process and accommodates for the interwoven contexts and processes.

For instance, immigrant adolescents undergo acculturation changes alongside possibly simultaneous developmental, biological, and social changes that affect their acculturation experiences (Titzmann & Jugert, 2015; Titzmann & Lee, 2018). Among the social changes is the aspect of inclusion or exclusion among peers. This is a common issue that arises for all adolescents (Ladd & Kochenderfer-Ladd, 2016) and, not surprisingly, research indicates it affects the acculturation process including the pace of acculturation (Riva & Eck, 2016). While short-term exclusion can lead to lack of belonging or finding meaningful existence in the host society, long-term exclusion may lead to feeling of alienation, depression, helplessness, and meaninglessness (Riva & Eck, 2016). Social exclusion and inclusion among immigrant youth are well documented and previous studies have shown that perceived discrimination by peers is a significant source of social exclusion, while social acceptance is associated with a sense of belonging in the host society, and leads to self-confidence (Mansouri & Kamp, 2007; Micklewright, 2002). This social aspect could underpin the motivation for acculturation among adolescents; acculturation could be viewed as means to achieve having friends, feeling included and having self-confidence, rather than a goal in itself.

An important arena for this social aspect of adolescence is school. Research indicates that school is a place where immigrant children can obtain support, recover from immigration stressors, improve their health, advance their acculturation and adjustment to the host country, and gain a sense of stability, social belonging, and respect (Fazel, 2002; Khawaja et al., 2018; Kia-Keating & Ellis, 2007; Walton & Cohen, 2011). A sense of belonging at school, ‘the extent to which students feel personally accepted, respected, and included by others in the school environment” (Goodenow, 1993, p. 80), has been found to improve adolescents’ well-being and social and academic development (Jose et al., 2012; Khawaja et al., 2018; Kia-Keating & Ellis, 2007). However, school can also be a place where immigrant adolescents might experience social exclusion and perceived discrimination, which can in turn affect their psychosocial and academic adjustment (Goodenow, 1993; Oxman-Martinez & Choi, 2014).

This central factor of social inclusion and exclusion is not limited to peers but also extends to the relationship with teachers. Teacher–student relationships and teachers’ support of and belief in students’ abilities have been found to be correlated with higher academic achievement (Kia-Keating & Ellis, 2007; Uslu & Gizir, 2016). Teachers’ support plays an important protective role in the psychological well-being of immigrant adolescents (Cristini et al., 2011). Adolescents who feel more connected to their teachers and school have been reported to be more motivated to attend school (Close & Solberg, 2008), which, in turn, has been associated with better academic achievement, higher self-efficacy levels, and less depression (Kia-Keating & Ellis, 2007). Regrettably, research indicates that immigrant adolescents have experienced perceived discrimination by teachers (Mansouri & Kamp, 2007; Oxman-Martinez & Choi, 2014). Family and specifically parents are, of course, not detached from this school inclusion phenomenon; parental support contributes positively to adolescents’ school belonging, psychological well-being and academic achievement (Trickett & Birman, 2005).

When considering the multifaceted nature of acculturation, one should not focus only on the receiving country but also be mindful of where the individual is migrating from, including the events and circumstances they may have been exposed to prior to migration. Pre-migration events, such as trauma exposure, separation from parents and age of immigration, are risk factors for poor mental health (Palmer & Ward, 2007; Steel et al., 1999). Not only is the home country important in terms of pre-migration factors, but also with regard to how it relates to the new country. The first, rather obvious, issue that can arise is a difference in spoken language. Language proficiency interacts with acculturation and has been shown to affect self-confidence (Clément, 1986) and identity (Noels et al., 1996). The degree of immersion into the new culture has been shown to affect language proficiency (Jiang et al., 2009). This implies the interaction between acculturation and language is bidirectional; language skills are required to acculturate, and acculturation is a means to enhance language skills. The cultural and ethnic distinctiveness of the immigrant group and the “cultural fit” between the immigrant and host populations may also be important (Crocker & Major, 1989). Highly visible and culturally distant groups may experience higher levels of discrimination. Yet, some research indicates that these highly visible groups possess the self-protecting function of externally attributing the discrimination to the home culture, not to them as a person (Crocker & Major, 1989; Liebkind & Jasinskaja-Lahti, 2000b). A further aspect to consider is the prevailing immigration and integration policies of the new country, which have been shown to greatly affect acculturation experiences (Berry, 1991; Berry and Kim, 1988; Murphy, 1965; Westin, 1991, 1993).

### Somali immigrant adolescents in Sweden

Somalis have migrated to Western countries due to a prolonged war and conflict in Somalia. Studies across various countries have reported that both Somali immigrant parents and children experience acculturation challenges even though the parents display a willingness and make efforts to transition to their new host country (Bowie et al., 2017; Degni et al., 2006; Nilsson et al., 2012; Osman et al., 2016). Yet, this body of research largely relies on parent-report. Explorations of Somali immigrant adolescents’ personal perspectives on acculturation are somewhat limited. First-hand accounts of Somali adolescent experiences in the USA (US) indicate acculturation problems and experiences that affected their mental health (Ellis et al., 2010, 2008). Another study, conducted in Finland, found that immigrant adolescents from Somalia and Russia experienced more perceived discrimination than immigrant adolescents from Vietnam and Turkey (Liebkind & Jasinskaja-Lahti, 2000a). Moreover, Somali adolescents were less acculturated than the other immigrant groups (Liebkind & Jasinskaja-Lahti, 2000a). Acculturation stressors reported by Somali immigrant adolescents in the US context include difficulties to adapt to the new culture and school system, as well as language barriers, social exclusion, and racial discrimination (Scuglik & Alarcon, 2005).

In 2017, Sweden received the highest per-capita number of immigrants and stateless people in Europe (Eurostat, 2018). The Somali population is the largest African immigrant group in Sweden. At the end of 2018, approximately 68,678 Somalis, 23% of whom were children younger than 18 years old, lived in Sweden (Statistics Sweden, 2018). Given this huge representation of Somali minors in Sweden, it is important to understand the acculturation experiences of this group to inform acceptable and effective integration strategies. The Somali culture is visible and culturally distant to that of Swedish culture; however, Sweden has a long-standing reputation for being among the most open and liberal countries with regard to migration (OECD, 2011). How this specific immigration context unfolds during the transitional stage of adolescence, given the aforementioned developmental and social processes that are at play, is underexplored.

The aforementioned literature on acculturation indicates that Somali adolescents in Sweden may experience social exclusion, particularly in the school setting with experiences of perceived discrimination not only from peers but potentially from teachers too. They may also experience difficultly regarding trauma exposure prior to migration, as well as pragmatic transition challenges, such as issues relating to language proficiency. Looking at other data sources within Sweden, a cultural clash in the school setting and high levels of discrimination appears likely.

In a previous Swedish study, Somali parents reported that their children had experienced acculturation problems, particularly in the school context as most had not received schooling in their home country due to the prolonged conflict (Osman et al., 2016). Furthermore, as newly arrived Somali pupils in the Swedish school system, they are subjected to an introductory system that is not well adapted to handle the differences that can co-exist between the different pupils regarding their former school experiences (Sharif, 2017; Skowronski, 2013). With regard to discrimination, a recently published report by BRÅ (2018), the Bureau of Crime Prevention, showed that xenophobic hate-crimes were four times more common against those with an African background. Persons with an immigrant background were also over-represented among victims of anti-religious hate crimes.

## Objective

The aim of this study was to investigate Somali immigrant adolescents’ experiences of social inclusion and exclusion as well as their need for support in their efforts to acculturate into society and the school environment. We sought to answer the following research questions: How do Somali immigrant adolescents experience social inclusion and exclusion in relation to Swedish society? What supports their acculturation into society and the school environment?

## Method

### Design

As a follow-up study in a larger project (Osman et al., 2017), an exploratory qualitative approach using focus group discussions (FGD; Creswell, 2014) was adopted to understand adolescents’ perspectives and experiences regarding their need for support and their acculturation in the new country. The FGD method enabled grasping this phenomenon, which is not well understood from the adolescents’ perspective (Green & Thorogood, 2014).

### Setting and participants

The study was conducted in a medium-sized city in Sweden (52,000 inhabitants), in which a high intake of refugees and immigrants in recent years has resulted in 19% of the population having an immigration status (Statistics Sweden, 2018). The city is historically an industrial community with a relatively high unemployment rate. In addition, there are a few vulnerable areas with a relatively high crime rate in which the majority of the Somali population lives. The research was part of a long-term follow-up study in which Somali-born parents involved in a culturally tailored parenting support programme were interviewed (Osman et al., 2019, 2017, 2016). In this study, we aimed to understand their children’s perspectives on their acculturation and what support they needed. Once the parents gave their consent and the contact information for their children, the adolescents were contacted by phone and informed about the purpose and procedures of the study. Those who were interested were provided with both written and oral information about the study and were invited to participate in an FGD.

Recruitment of participants continued until the saturation level for various experiences was reached or, in other words, no new conceptual information was forthcoming from the participants. In the initial plan, each family participating in the parenting support programme would send only one child to the FGDs, in particular, the child whom they had discussed in a previous study in the larger research project (Osman et al., 2017). However, in almost every FGD, siblings from the same family participated together.

Six FGDs with 47 adolescents (25 girls and 22 boys) were conducted. The age of the participants was between 14 years to 18 years, almost half of the participants had lived in Sweden for fewer than 5 years, and were in lower secondary school (see Table I).

**Table I.** Adolescents’ characteristics (n = 47)

Variable	Adolescents
	n (%)
**Adolescents**	
Boys	22 (47)
Girls	25 (53)
**Participants’ age, mean (SD)**	16 ± 2
**Time living in Sweden**	
1–5 years	23 (49)
6–9 years	10 (21)
≥ 10 years	14 (30)
**School level**	
Upper secondary	18 (38)
Lower secondary	28 (60)
Other	1 (2)

### Data collection

Data were collected using a semi-structured, pre-defined interview guide (see Table II), which was pilot tested with the first FGD with no further revisions required. The FGDs were carried out in both Somali and Swedish. As moderation language has been shown to affect the quality of qualitative research with ethnic minorities (Sills & Desai, 1996), all the adolescents were given the choice to speak the preferred language in which they felt comfortable sharing their thoughts and experiences. Two FGDs were conducted exclusively in Somali, two in Swedish, and two in a mixture of Swedish and Somali.

**Table II.** Topic guide for the focus group discussion

Topic	Questions
Acculturation process	Can you tell me or describe what it is like to be a youth or adolescent in Sweden? In your school, society and home? How would you describe your journey in Sweden so far? When thinking about acculturation and integration in school and society, what comes to your mind? What facilitates or hinders integration to Sweden? How have these challenges and opportunities affected you personally?
Acculturation support	How can one facilitate integration and acculturation to a new country? What would support acculturation into a new country? What kinds of strategies have you used?

The first and second authors moderated and observed the FGDs. The moderator’s role was to lead the discussion, ask probing questions, and ensure that all the participants had chances to share their experiences. The observer’s role was to take notes on how the group members interacted. The moderator and observer reflected on and discussed the outcomes of the discussions and their perceptions of the outcomes. These reflections and notes were taken into account during the analysis. Since the groups were constructed by convenience, there was some variation in group composition. Yet, the groups were heterogeneous, meaning they were mixed in terms of the age of the participants, the number of years participants were in Sweden, and gender (except two FGDs). Four FGDs were mixed with both girls and boys, and one FGD for girls and one FGD for boys. Between six and nine adolescents participated in each group. The group dynamics were largely positive. Almost all of the participants discussed the different topics in the interview guide; when some individuals were dominating the discussion, the moderator invited other participants to comment whilst being mindful not to control the discussion but creating space for every participant to contribute. The moderator was experienced in leading focus groups, which installed confidence in when to intervene and how to do so without affecting the group dynamic (Krueger, 1993). There were no apparent gender differences in how the discussions developed. However, in one group there were constant interruptions and distraction, which might be caused by an international sporting event that was occurring at the time. The themes were common across the FGDs.

Each FGD lasted 1–1.5 hours and was tape-recorded. The recording of the discussions supported the auditability of the research as it enabled the other researchers involved in the analysis to follow the decision trail (Beck, 1993). The adolescents were given gift cards worth 200 SEK (US$20) to compensate them for their time. They were not informed about the gift cards before the FGDs to avoid influencing them.

### Data analysis

All the FGD data were transcribed word for word and analysed inductively using Attride-Stirling’s (2001) thematic network analysis model. The analysis started with reading the transcripts several times to get an overview of the content. Meaningful text segments (sentences and paragraphs) capturing key concepts were highlighted and extracted into a table, which was used to devise a coding scheme to apply to the data. The codes explicitly focused on the core object of analysis: the adolescents’ thoughts and experiences on their acculturation and being young in Sweden. Themes were then abstracted from the coded text segments to grasp the underlying patterns. All the basic themes were merged and arranged into organizing themes that encapsulated and summarized the core of the basic themes. Finally, an overarching global theme (superordinate theme) capturing the principal metaphor in the text was identified.

The analysis was led by the first and second authors, who conducted the analysis separately. Both have Somali and Swedish language skills that could be utilized to capture the nuances of the language the participant described their experiences (Sills & Desai, 1996). Together with the other authors, the codes, themes, and organizing themes were reviewed. This improved the credibility of the research as it reduced the potential for researcher bias and selective inattention (Beck, 1993). The discussion resulted in the refinement and relabelling of basic themes and merging two proposed global themes into one. The findings were also presented and discussed with the study participants, which further enhanced credibility (Beck, 1993).

### Ethical considerations

Ethical approval for the study was obtained from the Regional Ethical Review Board in Uppsala, Sweden (DNR 2014/048/2). The adolescents and their parents were informed about the study’s purpose and procedure. Even though some adolescents were older than 15 years and could give consent to participate in the study, the parents wanted to give their consent before we contacted their children. This aligns with the previous hesitation for children to be interviewed in the previous study (Osman et al., 2017). The adolescents were informed orally and in writing that their participation was voluntary, and all the data would be kept confidential.

## Results

**Figure 1.**
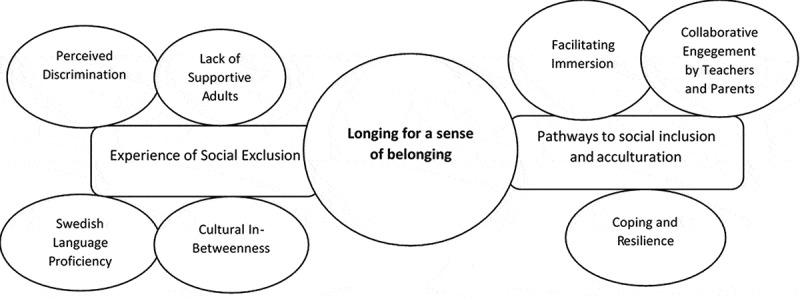
Somali adolescent’s experience of social exclusion and pathways to social inclusion and acculturation

One global theme emerged from the analysis: *Longing for a Sense of Belonging*. This concept related to the participants’ feelings of social exclusion and their efforts to acculturate into society. Two underpinning, organizing themes described the participants’ longing for a sense of belonging to society and school: *Experience of Social Exclusion* and *Pathways to Social Inclusion and Acculturation* (Figure 1).

### Experience of social exclusion

The first organizing theme described the participants’ experiences and challenges acculturating into the new country. Their acculturation efforts were hindered by their new school system, social exclusion, and a lack of support in coping with the transition. Four basic underlying themes explained the participants’ experiences of social exclusion: (i) *Perceived Discrimination*; (ii) *Lack of Supportive Adults*; (iii) *Swedish Language Proficiency*; and (iv) *Cultural In-betweenness*. All names in the focus group excerpts are fabricated.

#### Perceived discrimination

Many immigrant Somali adolescents reported discrimination that manifested in a range of forms. The participants described encountering racism and discrimination based on their ethnicity, religion, and skin colour in public places, society, school and in their searches for summer jobs. All participants stated that these experiences of discrimination posed barriers to acculturation in the new country and left them feeling excluded, vulnerable, and sometimes angry.

Participants encountered many different types of discrimination and the school setting figured prominently. Participants described the discrimination in school as teachers’ low expectations of their capacity and ability to manage school. They also perceived discouragement from teachers and unequal treatment of native and immigrant students. When asked to elaborate on how they experienced perceived discrimination from teachers, participants described both direct and indirect forms. Direct discrimination was based on the immigrants’ religion and how they dressed.

*Faiza: There are a lot of teachers who joke about the girls’ veils and ask why they do not take off their veils. They say, for instance, “Isn’t it warm?” They ask all those stupid questions, and then everybody looks at [the girls] and laughs.’*

In another example of direct discrimination, a participant reported that she always had to submit her completed tasks to a plagiarism-checker, while her friends did not have to do so.

Regarding experiences of indirect discrimination, the Somali adolescents in all the FGDs emphasized that their teachers had low expectations of them and did not trust their capacity to learn. The participants found that even if they worked hard, their teachers were surprised and lacked faith in their ability to do well.

*Moderator:* *Can you give an example when you felt discriminated by your teacher/s?*

*Hafsa:* *If you wrote everything correctly in your task and the exam, the teachers would not believe that you did it, and they think that someone else helped you.*

*Sara:* *[The] same [thing] happened to me. My classmates and I did a group project, and I worked hard with them. We were supposed to get the grade as a group, but the other girls got an A and I got a C, and when I tried to argue, the teacher said, “I am your teacher and know best.” That is unfair.’*

Several participants stressed that the teachers and study counsellors discouraged their future plans and dreams to become doctors, engineers, and other professionals.

*Nasra:* *My teacher asked us about our future plans; we were four girls. I said, “I want to become an optician,” and he said, “That will never happen.” Then he asked another girl, who was Swedish, who said that she wanted to become a veterinarian, and he said, “That is possible.”*

*Ahmed:* *I went to this study counsellor to apply for upper secondary school. He asked me what my future plans were. I told him, “I want to go to university and become an engineer.” He said, “That is not possible; it is easier if you apply for vocational education.”*

Many participants stated that stress and low self-confidence resulted from the perceived discrimination of teachers, while teachers’ low expectations led to the participants’ feelings of discouragement and hopelessness. The adolescents also noted that such stress and hopelessness might have led some of their friends to drop out of school and engage in poor behaviour. Although many participants emphasized perceived discrimination by teachers, a few who had arrived in Sweden at very young ages did not have similar experiences.

#### Lack of supportive adults

Many immigrant Somali adolescents reported a lack of parental and social support, as well as a lack of hobbies and role models, which contributed to their difficulties with acculturation. Some participants explained that they lacked parental support, as their parents were not proficient in Swedish, and some parents had minimal levels of education. Their parents were unfamiliar with the Swedish school system and its administrative procedures.

*Zahra:* *We have this admin system in the school in which parents can check the curriculum and assignments in school and send notifications if their children are sick. My mom couldn’t understand [it]. I took the responsibility to show her and send a notification if I was sick.*

*Nasra:* *You know, some could deceive their parents and send a notification that they are sick and tell their parents that they are off from school.*

Many participants also reported that their parents had unrealistic expectations about their academic achievements and became disappointed if they failed subjects. *“It is kind of the Somali culture to yell at children when they earn a grade of F, [parents] expect you to be mentally strong.”*

Many participants had strong opinions on the lack of role models and supportive adults. “*There are not so many adults who are involved in the lives of the youth,”* said one participant. *“If you have dreams, there is no one who can guide you.”*

Some participants emphasized the importance of access to counselling centres that could provide support when no trusted adult was available.

*Moderator:* *What kind of support do you need and who can support you?*

*Zahra:* *Sometimes, you need an adult person outside the family who listens and does not judge.*

*Moderator:* *Don’t you have school counsellors who you can go to?*

*Zahra:* *I don’t know how the school counsellors can help us with the issues we have.*

*Basma:* *I agree, but there is other support in the society, which we can seek*

#### Swedish language proficiency

Many immigrant Somali adolescents highlighted the challenges they encountered in the new country that hindered their acculturation efforts, such as struggles adjusting to school due to inadequate language proficiency and feelings of hopelessness and despair. Many experienced a paradox while acculturating into the new country: to navigate it, they needed language proficiency, but they perceived Swedish society as exclusionary, making it difficult for newcomers to integrate and learn the language through social interactions.

*Moderator:* *What it is like to be a youth or adolescent in Sweden?*

*Hamda:* *Living in this society is both easy and difficult, if you don’t know the language it is difficult, and if you are born here and have social network it is easy.*

*Asha:* *To integrate into [Swedish] society is difficult because nobody talks to you, and you cannot take the initiative because you do not know the language.*

The participants expressed difficulties adjusting to school, keeping up with lessons and grasping school subjects due to their poor language proficiency. Some adolescents reported that they had not attended school in their home country, which made understanding the subjects even more difficult. When asked how these challenges affected them, almost all the participants stated that the challenges became sources of stress that caused feelings of hopelessness, which in turn led to a lack of confidence. Some also reported that their friends had dropped out of school and become involved in substance abuse.

*Moderator:* *What do you think caused your friends drop-outs or involvement in substance abuse?*

*Jamal:* *They could not manage their challenges and difficulties in the new country, and they might have thought that they had no other choice than to drop out.*

*Jamila:* *But I think it is not only the challenges of the society that made them drop-out or give up, my opinion is that they couldn’t manage different school subjects when they do not know the Swedish language adequately.*

Although most participants stressed the transitional challenges in the new country, a few who had come to Sweden at very young ages reported fewer transitional difficulties.

*Muna:* *I was four or five years old when I came to Sweden, so I started preschool here and learned the language more easily.*

*Abdi:* *Good for you. That is why you haven’t experienced what we did.*

#### Cultural in-betweenness

In four FGDs, the Somali immigrant adolescents reported that they felt as if they were between two cultures and not connected to either Somalia or Sweden. They related this cultural in-betweenness to their appearance, clothing, and behaviour that did not follow Swedish social codes. They explained that they were not accepted in Swedish society, but if they returned to their home country, they would no longer be considered Somali but instead people of the “diaspora.” “*I do not know which country I belong to or which culture I have,”* said one participant. *“I did not grow up in Somalia, and here, I do not celebrate the Swedish midsummer.”* Several participants reported they had one culture with their families and another culture outside their home, which precipitated what they called an “identity crisis.” In some FGDs, the participants who had recently arrived in Sweden expressed pride in being Somali. They emphasized the importance of maintaining their culture while at the same time adapting to Swedish culture.

### Pathways to social inclusion and acculturation

The second organizing theme related to the participants’ desire to feel as though they were a part of society and the factors they believed could facilitate their acculturation. Three basic underlying themes emerged from this organizing theme: (i) *Facilitating Immersion*; (ii) *Collaborative Engagement by Teachers and Parents*; and (ii) *Coping and Resilience.*

#### Facilitating immersion

Many Somali immigrant adolescents expressed a desire to adapt to the new country and offered various suggestions on how to achieve this goal. Some participants believed that they would find social and cultural adaptation easier in schools with more native children, as they normally had contact only with other immigrant children, which made social and cultural adaptation difficult. School, therefore, was viewed as an important arena that could facilitate acculturation between native and immigrant youths and support immigrant children’s adaptation. Most participants emphasized the bidirectional nature of acculturation.

*Moderator:* *You told me many of the challenges you have, but how can the integration be facilitated?*

*Ali:* *Integration is not only about immigrant youths integrating into native groups; both should try to understand each other, and school is a good place to do that.’*

*Hamda:* *but we are the one who came to this country, and we should try to integrate, isn’t it?*

Some participants suggested that social inclusion could be promoted through activities during school and after hours. Different kinds of sports and language-learning opportunities were seen as activities that could support acculturation and social adaptation.

*Abdi:* *Through activities such as sports, you learn how to adapt to society. Playing football with native youths in schools helps you get to know them and make friends, which, in turn, helps you feel happy and able to manage every hardship.’*

Many participants also desired support from society, particularly acceptance and inclusion, to facilitate their social adaptation. *“If society accepts you,”* said one participant, *“you feel included and can contribute to positive things.”*

#### Collaborative engagement by teachers and parents

All participants emphasized that support and motivation from teachers were crucial to newcomers’ school adjustment and overall well-being. They acknowledged the support and encouragement they received from language teachers and native teachers with competence in supporting immigrant children.

*Basma:* *My language teacher from Somalia encouraged me and said, “While other students are walking, you have to run [make double the effort] because you have educational opportunities in this country.”*

Many participants considered it important for teachers to have knowledge of immigrant children’s educational needs. The participants experienced stress and pressure from teachers who understood neither what they had gone through nor the difficulties they encountered in social and school adjustment.

*Moderator:* *You have told me that you experienced discrimination from your teachers? What can the school do? What can your teachers do to support you?*

*Nasra:* *I think that a more competent and knowledgeable teacher can help you to adjust, do you understand? It is important to have a teacher who thinks more of you as a human being.*

*Liibaan:* *… I would say that the teacher should look to the student’s knowledge and not only their language skills. For instance, if it is a maths problem solving, one student might know how to solve it in numbers but cannot tell you in words, so the teacher should judge by their knowledge and not this one’s name is Andersson and the other is Mohamed.*

Family support for youths’ school adjustment was regarded as the most important aspect of successful acculturation. Even though many participants reported that they could count on their families’ support, they wanted their parents to be more engaged in their schooling and to motivate them. Several participants emphasized the importance of collaboration among immigrant parents, school, and society to promote immigrant children’s health and well-being.

#### Coping and resilience

Many immigrant Somali adolescents described the strategies they used to manage and cope with the challenges they faced in feeling included in both school and society. For example, many of them accepted that there would be challenges in adapting to a new country.

*Jamila:* *There are challenges and changes in your life, you have to accept these because you have come to a new country, and everything is new to you. Eventually, you will adapt to it.*

Several participants stated that they had to tolerate discrimination in the host country, but they also emphasized that they should not give up on their goals. Some participants considered the setting of goals as a way to overcome these challenges. They believed that they would face problems anywhere, so they had to learn how to move on and not look back. In the FGDs, the participants repeated several times that having hope helped them cope.

*Moderator:* *What do you do to cope the difficulties?*

*Asha:* *Having hope is important, as well as not giving up. As time passes, everything will be sorted out for you.*

*Jamila:* *Don’t give up, you will meet other people who will encourage you … people are not the same …*

*Zahra:* *Understand and accept the change because you are in a new environment, new country, new people, everything is new that is why.*

Many participants stressed self-reliance and the taking of responsibility as helpful in dealing with their situations. They believed that they had the responsibility to acculturate into society, and they experienced self-reliance as their remaining support in the absence of other forms of support from school, society, and families. Some participants also cited religion and good, reliable friends as sources of support. All participants expressed gratitude for the opportunities given by the new country and their parents, which they contrasted with their possible circumstances if they had remained in war-torn Somalia.

*Jamila:* *[Our parents] left their home country for us to get better opportunities. … In Sweden, you have free education, free healthcare, so it is good here.’*

## Discussion

This study has investigated Somali immigrant adolescents’ experiences of social inclusion and exclusion as well as their need for support in their efforts to acculturate into Swedish society and the school environment. The adolescents had the motivation to acculturate but felt that obstacles hindered their acculturation, which, according to Titzmann and Lee (2018), could lead to the opposite result and set back acculturation. As anticipated, the adolescents raised the issue of perceived discrimination. They also revealed a lack of social support from parents or other adults in the community. Not surprisingly, Swedish language proficiency was raised as an issue. In addition, they spoke of the feeling of cultural in-betweenness. The respondents articulated a desire for immersion into the new culture and structured ways to achieve this. They emphasized the importance of optimized support for school adjustment, which was centred on a greater collaborative effort between teachers and parents. They also described a number of coping strategies they utilized to build personal resilience during the acculturation process.

Given the previous reports of immigrant adolescents having experienced perceived discrimination by teachers (Mansouri & Kamp, 2007; Oxman-Martinez & Choi, 2014), the high visibility of Somali culture in the Swedish context (Crocker & Major, 1989) and Swedish crime statistics indicating elevated levels of hate crime towards immigrants from African background (BRÅ, 2018), it is not surprising that perceived discrimination was raised in the present study. Regrettably, this appears to be a key acculturative stress for adolescents. The youth indicated the perceived discrimination had adverse effects on their mental health, which aligns with previous research (Ellis et al., 2010, 2008). Perceived discrimination can also contribute to poor school performance and a lack of school belonging (Stone & Han, 2005; Verkuyten & Brug, 2003).

Although championed by the Somali immigrant adolescents, it is not always possible to provide native teachers to support immigrant students. Yet, efforts can be made to improve the cultural awareness of other school personnel through dedicated training. Previous research indicates that students achieve at higher rates when their teachers are culturally sensitive, utilize culturally relevant teaching methods, and can create a genuinely empathetic relationship with youth who are culturally different (Gay, 2000; Howard, 2001; Ladson-Billings, 1994). Positive relationships between teachers and immigrant students have been reported to promote social connections (Thijs & Verkuyten, 2008).

The adolescents observed a disconnection between parents and school. It was presented as a dilemma regarding their parents’ unrealistic high expectations on academic achievement compared to teachers’ low expectations, which caused them stress. As we know from the literature, parental support contributes positively to adolescents’ school belonging, psychological well-being and academic achievement (Trickett & Birman, 2005). Thus, the consequential recommendation from the adolescents to promote a greater collaborative effort between parents and teachers to improve newcomer student adjustment to school is astute.

Our interviews do not allow us to ascertain whether or not teachers actually have low expectations and negative views on immigrant adolescents’ future potential, but it is probably safe to conclude that improved communication between the home and school seems necessary in the case of Somali adolescents. According to Bunar (2016), what contributes to open school culture is the respect and commitment for the differences, mutual respect, high expectation, and motivation. Parents and teachers are potent protective factors that can enhance students’ sense of school belonging and social inclusion; therefore, it is important to achieve a balance between demand and support through more effective communication between the school and the parents (Arslan, 2018; Bunar, 2016; Sellers & Shelton, 2003). This aligns with findings from Somali parents in Sweden, who have expressed negativity towards the relationship with their children’s schools (Osman et al., 2016). However, there was no apparent awareness of the high educational expectations placed on children by Somali parents (Osman et al., 2016).

Yet, a barrier to parents enacting the recommendation of improved collaboration is Swedish language proficiency. The language was identified as a barrier for parents, both by participants in the present study and by Somali parents themselves in a previous study (Osman et al., 2016), but also for the adolescents. They reported that language difficulties adversely affected their school adjustment and school belonging, which, in turn, led to stress and lower self-confidence. This was another anticipated finding. We know from the literature that language proficiency interacts with acculturation and has been shown to affect self-confidence (Clément, 1986) and identity (Noels et al., 1996). What is interesting is the adolescents’ ability to recognize the value of immersion. The youth desired inter-ethnic relationships with native adolescents and, to promote their language development, acculturation, and social inclusion, suggested arranging sport and learning activities for both immigrant and native children during and outside schooltime, which is advocated by many researchers (e.g., Borsch et al., 2019; Fazel et al., 2012; Pastoor, 2015). Cultural immersion has been linked to enhanced language proficiency (Jiang et al., 2009) and organizing structured events, such as the adolescents suggest, between socially distant groups is a widely adopted strategy used to promote social cohesion among children and adolescents (Ladd & Kochenderfer-Ladd, 2016). Moreover, Kim et al. (2019) suggest that social support, activities, and community programmes can promote immigrant adolescent’s coping strategies and a sense of belonging. Thus, the adolescents’ suggestion demonstrates high levels of insight and pragmatism.

To foster the development of immigrant children’s language proficiency (Close & Solberg, 2008; Schachner et al., 2017; Wang & Holcombe, 2010), schools need to construct an inclusive, supportive environment in which the individual needs of each immigrant student are considered (Bunar, 2016; Sharif, 2017; Skowronski, 2013). Schools should employ a holistic support approach building on children’s strengths, previous education, and networks and support in school and society (Schachner et al., 2017, 2018; Sharif, 2017; Skowronski, 2013). Skowronski (2013) argued that the schools and the classrooms to be more permissive atmosphere in which language can be used in different and not be ashamed when one is not fluent in the native language because this will give newcomers as well as pupils who do not have the native language as their first language to feel included.

## Conclusions

The findings from this study, although undesirable in that the adolescents disclosed challenges and stress related to acculturation, are encouraging in that the youth identified pragmatic solutions in line with wider beliefs about potentially effective integration strategies. Somali immigrant adolescents in Sweden appear to seek structured integration opportunities, promote stronger efforts from schools to connect with their parents and recognize that language proficiency underpins the success of these methods. Not only does this show the aptitude of young people to inform policies that relate to their daily lives, it suggests that if such policies were pursued they would be met with acceptance, which would aid success.
